# Exploration of Agonist and Antagonist Binding Sites within the Cytosolic AHR Complex Using Molecular Modeling

**DOI:** 10.1021/acsomega.5c10598

**Published:** 2026-03-05

**Authors:** Ivana Karabogdan, Francisco Yanqui-Rivera, Deepak Sayeeram, Ahmed Sadik, Aubry K. Miller, Saskia Trump, Ute F. Röhrig, Christiane A. Opitz

**Affiliations:** † German Cancer Research Center (DKFZ), Heidelberg, Division of Metabolic Crosstalk in Cancer and the German Cancer Consortium (DKTK), 28333 DKFZ Core Center Heidelberg, 69120 Heidelberg, Germany; ‡ Faculty of Bioscience, Heidelberg University, 69120 Heidelberg, Germany; § Cancer Drug Development, German Cancer Research Center (DKFZ), 69120 Heidelberg, Germany; ∥ Molecular Epidemiology Unit, Berlin Institute of Health at Charité and the German Cancer Consortium (DKTK), 522475 Partner Site Berlin, a partnership between DKFZ and Charité -Universitätsmedizin Berlin, 10117 Berlin, Germany; ⊥ Molecular Modelling Group, 30489 SIB Swiss Institute of Bioinformatics, CH-1015 Lausanne, Switzerland; # Neurology Clinic and National Center for Tumor Diseases, 69120 Heidelberg, Germany

## Abstract

The aryl hydrocarbon receptor (AHR) is a ligand-activated transcription factor involved in metabolism, cell motility, development, and immune responses. Its dysregulation is linked to various diseases, including cancer, in which it can enhance tumor progression and suppress immune responses. High-resolution cryo-electron microscopy (cryo-EM) structures of the human cytosolic AHR complex have recently been solved and have provided insights into its agonist-binding mechanisms. However, our understanding of AHR antagonist binding remains limited. Our computational study, using the structure of the indirubin-bound human cytosolic AHR complex together with state-of-the-art docking algorithms and molecular dynamics simulations, suggests that AHR antagonists may bind either to the ligand-binding pocket or to alternative, as yet unexplored, sites outside of the ligand-binding pocket. These findings suggest novel molecular mechanisms of AHR inhibition and provide the foundation for experimental evaluation to advance our understanding of the therapeutic potential of current AHR inhibitors and to support future drug development efforts.

## Graphical abstract

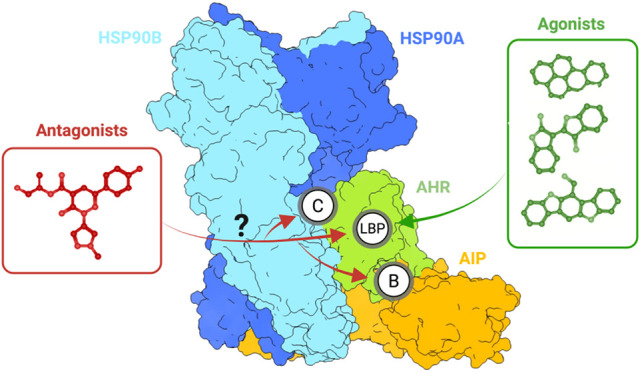

## 1 Introduction

In recent years, the transcription factor aryl hydrocarbon receptor (AHR) has emerged as an important sensor of compounds derived from various sources such as the environment, diet, and endogenous metabolites.^1,2^ Numerous compounds representing ligands of the AHR, exerting either agonistic or antagonistic functions, have been identified to date. The AHR is involved in a plethora of biological and physiological processes, including drug or xenobiotic metabolism, barrier integrity, cell proliferation and migration, immune modulation, inflammation, infection, and cancer.^3–12^ AHR belongs to the basic helix–loop–helix (bHLH) and periodic circadian protein (PER)—AHR nuclear translocator (ARNT) single-minded protein (SIM) [PAS] superfamily of transcription factors.^13,14^ Its structure can be subdivided into specific regions with distinct biological functions: (1) the N-terminal bHLH domain that is mainly involved in DNA recognition and dimerization, (2) the two PAS domains, A and B, that are important for protein heterodimerization and ligand binding, respectively, and (3) the mostly unstructured C-terminal transactivation domain.^13,15^ In its inactive form, AHR is localized in the cytoplasm, forming a complex with two heat shock proteins 90 (HSP90), AHR-interacting protein (AIP, formerly XAP2), and prostaglandin E synthase 3 (PTGES3, formerly p23).^16^ Further, the nonreceptor tyrosine kinase SRC has been identified as part of the cytosolic AHR complex in different cell types.^17–19^ Binding of an agonist to AHR results in activation of the transcription factor, inducing its translocation from the cytoplasm to the nucleus and heterodimerization with ARNT.^20–23^ Binding of the AHR-ARNT complex to specific DNA recognition sites controls the expression of a myriad of genes.^24,25^

When dysregulated, AHR is implicated in many diseases, including cancer, inflammation, and immune diseases.^9,12,26–32^ In multiple cancers, AHR expression is elevated and AHR activation is mediated through the generation of endogenous ligands, thereby enhancing tumor cell intrinsic malignant properties, including cancer cell proliferation and migration, and suppressing antitumor immune responses.^12,33–37^ However, in line with its context specificity, AHR can also exert tumor-suppressing functions.^38–40^ Nonetheless, AHR is considered a potential therapeutic target, which has driven the development of drugs targeting AHR. From the limited number of compounds that have progressed to clinical trials, BAY2416964 showed potent and selective AHR antagonism, restoring immune function and enhancing cytotoxic T-cell activity in vitro. Moreover, it was well-tolerated and demonstrated antitumor effects in mouse cancer models.^41^ A better understanding of the binding mode of AHR-inhibiting compounds such as BAY2416964 is of great significance in advancing our knowledge of how to efficiently target the AHR and uncover the true therapeutic potential of AHR-targeting drugs.

Numerous efforts have been made to unravel the mode of ligand binding to the cytosolic AHR complex through structural analyses and modeling.^42^ The first crystal structure of the PAS-A domain from murine AHR provided initial insights into the PAS-A and bHLH domains.^43^ Later, the bHLH PAS-A heterodimer structure of human AHR and murine ARNT was determined.^44,45^ Over many years, the PAS-B domain—the orthosteric ligand-binding site—remained structurally unresolved due to expression and solubility challenges.^45^ Whereas computational modeling of the PAS-B domain of the AHR and application of computer-aided drug discovery methods have offered relevant insights about the transcription factor’s ligand-binding properties,^46–51^ the absence of an experimental PAS-B structure limited the precise understanding of the molecular mechanism of ligand interactions. Only recently, high-resolution cryo-electron microscopy (cryo-EM) structures of the human AHR cytosolic complex containing the cochaperones HSP90 and AIP, and including an indirubin- or benzo­[*a*]­pyrene (B­[*a*]­P)-bound AHR PAS-B domain, were revealed, providing the opportunity of exploring ligand-specific binding modes.^52,53^ In addition, Wen and colleagues uncovered the murine AHR cytosolic complex with bound PTGES3, discussing the role of the chaperones in stabilizing and activating the AHR.^54^ The most recent structural insight into the confirmation of the multidomain AHR-ARNT-DNA complex containing both PAS-A and PAS-B domains has provided a mechanistic explanation for how AHR transitions from the cytosolic to the nuclear complex.^55^

These structures present an opportunity to investigate the molecular details of ligand binding. For this purpose, we employed the indirubin-bound cytosolic AHR complex as a target structure. Global docking across the entire surface of the AHR complex with the state-of-the-art algorithm Attracting Cavities (AC)^56,57^ was used to explore the binding of chemically diverse compounds, including agonists and antagonists. To gain further insights, local dockings within identified binding sites were carried out both with AC as well as AutoDock Vina (Vina),^58,59^ one of the most extensively used algorithms in structure-based drug discovery. Molecular dynamics (MD) simulations of relevant docking poses of an agonist and an antagonist in different binding sites provided additional insight into their interactions, stabilities, and influences on the AHR complex structure. The findings of our molecular modeling study suggest that (1) AHR antagonists may bind to the ligand-binding pocket (LBP) of the AHR, inducing a structural rearrangement, and (2) AHR antagonists may bind to two alternative sites located at the interface of the AHR and AIP, or the AHR and both HSP90 molecules, respectively. An orthogonal pocket detection tool, Fpocket,^60^ substantiated our assumptions regarding the possibility of novel antagonist binding sites within the cytosolic AHR complex. *In silico* alanine mutation of the AHR complex suggests potential target residues for the experimental validation of the proposed allosteric binding sites. Our study contributes to a better understanding of AHR antagonist binding, which could offer new avenues for future drug development.

## 2 Methodology

### 2.1 Preparation of the Target Structure

The cryo-EM structure of the indirubin-bound AHR complex (Protein Data Bank (PDB) ID: 7ZUB)^52^ was retrieved from the PDB (RCSB.org).^61^ The target structure was prepared using the “Dock Prep” utility of UCSF ChimeraX,^62–64^ deleting alternate locations and reconstructing incomplete side chains with the Dunbrack rotamer library.^65^ For the modeling, the ADP/molybdate cofactors were replaced by adenosine triphosphate (ATP) to reflect physiologically relevant HSP90 states, and indirubin was removed. Histidines were protonated according to their environment, and missing hydrogen atoms were added using the HBUILD command of CHARMM.^66^

### 2.2 Molecular Docking

#### 2.2.1 Docking with Attracting Cavities

Ligands as well as ATP topologies and parameters were generated with the Merck Molecular Force Field (MMFF)-like approach of SwissParam.^67^ Ligands were considered in their standard protonation state at pH 7, except for kynurenic acid (KynA), where the neutral species was docked. We used the CHARMM36 force field for the proteins and the CHARMM program, version c48b1. The AC scoring function corresponds to the total CHARMM energy, including the Fast Analytical Continuum Treatment of Solvation (FACTS) implicit solvation terms.^57,68^

For global (blind) docking, attractive cavity points were generated for the cytosolic AHR complex, containing AHR amino acids 271–427, with a default N_Thr_ value of 70. To focus the search on AHR interaction sites, only points within a maximal distance of 10 Å to any atom of the AHR were retained, yielding a total of 304 attractive points. The default initial ligand rotation angle of 90° was chosen, and no random initial conditions (RIC) were applied to keep execution times reasonable.^57^ Subsequently, local docking calculations for the 3 identified potential ligand-binding sites were carried out, using a cubic search box with an edge length of 20 Å around their respective centers (LBP: 161, 164, 160; Site B: 151, 179, 144; Site C: 174, 173, 177), a N_Thr_ value of 50 to place attractive points also in shallow cavities, an initial ligand rotation angle of 90°, and 4 RIC. For analysis, we merged the local docking results and kept poses with a maximum relative score of 2 kcal/mol compared to the ligand conformation with the best score. For local docking with a flexible protein environment, all residues with at least one atom within up to 3.5 Å of a ligand pose were considered to be flexible. The same parameters as for rigid local dockings were used, except for a N_Thr_ value of 60 to decrease execution times.

To calculate Root mean square deviation (RMSD) values between docked and experimentally resolved ligand conformations, complex structures were aligned in ChimeraX using the match tool and the AHR PAS-B domain as a reference. Ligand coordinates were exported in MOL2 format and subsequently converted to SDF format with Open Babel (http://openbabel.org, v.3.1.1).^69^ Symmetry-aware RMSD calculations were carried out in Python (v.3.13.7) using RDKit (v.2025.03.6) in combination with spyrmsd (v.0.9.0).

If not stated otherwise, the conformation with the lowest AC score was used for graphical representation of the target structure (see “Section 2.1”) and the docked compounds (see “Section 2.6”).

#### 2.2.2 Docking with AutoDock Vina

For comparison, we performed local docking with the same target and ligands using AutoDock Vina (Vina), version 1.2.3.^58,59^ Vina is not suitable for blind docking, but to obtain a good sampling for each site, a cubic search box with an edge length of 25 Å and an exhaustiveness value of 100 was used. If not stated otherwise, the conformation with the lowest Vina score was used for graphical representation of the docked compounds (see “Section 2.6”).

### 2.3 Ligand-Binding Site Prediction and Characterization

To identify potential ligand-binding sites in the AHR complex, we utilized the prepared target structure (see “Section 2.1”) in conjunction with the Fpocket^60^ tool.

### 2.4 Performance and Analysis of MD Simulations

Periodic-boundary MD simulations of the AHR complex with different ligands were performed with Gromacs^70–73^ version 2023.3 (source code: 10.5281/zenodo.10017686; documentation: 10.5281/zenodo.10017699) using the CHARMM36 force field (charmm36-jul2022; https://mackerell.umaryland.edu/charmm_ff.shtml#gromacs)^74^ and the TIP3P water model.^75^ The MD simulations were run on a single thread of an AMD EPYC 7742 (or 7763) CPU core and one NVIDIA A100-SXM4-(or -PCIE)-40GB GPU with an average performance of 0.9 h/ns. Electrostatic interactions were computed with the Ewald particle-mesh method with a grid spacing of 1.2 Å and a fourth-order spline interpolation. A cutoff of 1 nm was applied for the real-space direct sum part of the Ewald sum. van der Waals (vdW) interactions were included up to 1 nm and smoothly switched to zero from 1 to 1.2 nm. Bonds involving hydrogen atoms were constrained by using the P-LINCS algorithm.^100^ The systems were coupled to an isotropic C-rescale barostat^76^ with a coupling constant of 1 ps and a compressibility of 4.5 × 10^–5^ bar^–1^. The solute and the solvent were separately coupled to two V-rescale thermostats^77^ with a relaxation time of 0.2 ps. The time integration step was set to 2 fs, the temperature to 300 K, and the pressure to 1 bar. A cubic simulation cell with an edge length of 16 nm was used to prevent direct interactions between periodic images, resulting in about 400,000 atoms per system. Sodium and chloride atoms were added to neutralize each system and to obtain a physiological salt concentration of 150 mM. Initial atom coordinates were taken from the AC docking results, optimized by 500 steps of steepest descent, heated from 0 to 300 K over a period of 1 ns, equilibrated for a further 9 ns, restraining each solute non-hydrogen atom to its original position, and finally equilibrated for 10 ns without restraints before starting data collection over 200 ns. For each system, 8 replicates with different initial velocities were simulated for a total of 1.6 μs of production time, with coordinates saved every 0.01 ns.

Five systems were investigated: (1) ligand-free (Apo), (2) indirubin bound within the LBP (Indirubin_LBP_), (3) BAY2416964 bound within the LBP (BAY2416964_LBP_), (4) BAY2416964 bound to site B (BAY2416964_Site B_), and (5) BAY2416964 bound to site C (BAY2416964_Site C_). The starting structure for system (2) was based on the experimental structure, that for system (4) on rigid local docking, and those for systems (3) and (5) were taken from local docking with a flexible protein environment (see “Section 2.2.1”). Prior to analysis, structural snapshots were superimposed using all protein backbone atoms, excluding AIP residues 2–165 due to their high flexibility.

Clustering analysis of the ligands, indirubin and BAY2416964, was performed with the Gromos algorithm^78^ in gmx cluster with a cutoff of 0.3 nm, considering ligand heavy atoms and structural snapshots extracted every 0.5 ns. To cluster the PAS-B domain (AHR residues 286–387), structural snapshots were superimposed, considering only the backbone of these residues. Clustering of the protein heavy atoms was performed using the same parameters as above, except for a cutoff of 0.25 nm.

The command gmx hbond was applied to structural snapshots extracted every 0.05 ns to extract information on hydrogen bonds throughout the simulations. For the graphical representation, only hydrogen bonds established with a frequency of 30% or more were considered.

Nonbonded interaction energies were analyzed by identifying residues in contact with a ligand or a residue of interest, defined as having a minimum distance below 0.42 nm for at least 30% of the simulation. The gmx pairdist tool was used for determining the contact residues. The selected residues were then subjected to analysis using gmx energy. For graphical representation, only nonbonded interactions with energies less than or equal to −10 kJ/mol in at least one of the replicates were considered. Equivalently, contact residues and their nonbonded interaction energies with residues of interest were determined.

Root mean square fluctuations (RMSF) and RMSD were calculated using all protein backbone atoms except residues 2–165 of AIP. RMSF and RMSD values for each residue of the AHR backbone were calculated with the tool gmx rmsf separately for each MD replicate before averaging. For assessment of the conformational stability of the ligands, the tool gmx rms was used, calculating the RMSD of the ligands as a function of time over all replicates.

### 2.5 *In Silico* Alanine Scanning

*In silico* alanine scanning was carried out with FoldX version 5.1 (https://foldxsuite.crg.eu/). For this purpose, structural snapshots of the MD simulations of the three systems BAY2416964_LBP_, BAY2416964_Site B_, and BAY2416964_Site C_ were extracted every 1 ns and subjected to AlaScan with default parameters, yielding the Gibbs free energy ΔG for mutating each amino acid to alanine. A ΔG value below 6 kJ/mol was interpreted as indicating that the corresponding mutation would have only a limited impact on the structural integrity of the complex.

### 2.6 Figure Preparation

ChemDraw (v.21.0.0.28, v.23.0.1.10) was used to draw the molecular structures of the agonists and antagonists. All figures of structural models were generated using ChimeraX v1.8.1,^62–64^ using the swissdock format for visualizing docking poses, and the commands hbonds and contacts for analysis of hydrogen bond formation and vdW contacts, respectively. Data analysis was performed using R Statistical Software (v.4.3.3) (https://cran.r-project.org/) and Bioconductor (v.3.18) (https://bioconductor.org/). The base packages base, stats, utils, and grDevices, as well as the packages purrr (v.1.0.2), tibble (v.3.2.1), stringr (v.1.5.1), ggplot2 (v.3.5.1), Peptides (v.2.4.6), tidyr (v.1.3.1), readxl (v.1.4.3), and tools (v.4.3.3) were used for data processing and generation of graphical depictions. The RMSD and RMSF plots were generated using gnuplot (v.5.4 patchlevel 2). The graphical abstract was generated using BioRender (https://www.biorender.com/).

## 3 Results and Discussion

The recent cryo-EM structure of the indirubin-bound human HSP90-AIP-AHR cytosolic complex^52^ provides the unique opportunity to investigate ligand binding and unveil the binding mechanisms of established AHR agonists and antagonists. Such insights are pivotal to enhancing our understanding of molecular mechanisms that regulate AHR activity. In this study, we focused on a selection of eight AHR agonists and five AHR antagonists (Figure 1). The agonists represent well-established activators of the AHR that are either indole-based compounds typically derived from tryptophan, including indirubin, 6-formylindolo­[3,2-*b*]­carbazole (FICZ), methyl-2-(1H-indole-3-carbonyl)­thiazole-4-carboxylate (ITE), KynA, indole-3-acetaldehyde (I3A), and indole-3-carboxaldehyde (I3C), or xenobiotic agonists such as benzo­[*a*]­pyrene (B­[*a*]­P) and β-naphthoflavone (BNF).^79–82^ The AHR antagonists BAY2416964, KYN-101, GNF-351, SR1, and CH223191 were selected because of their well-studied ability to inhibit AHR activity and their application in preclinical or clinical studies.^41,83,84^

**Figure 1:**
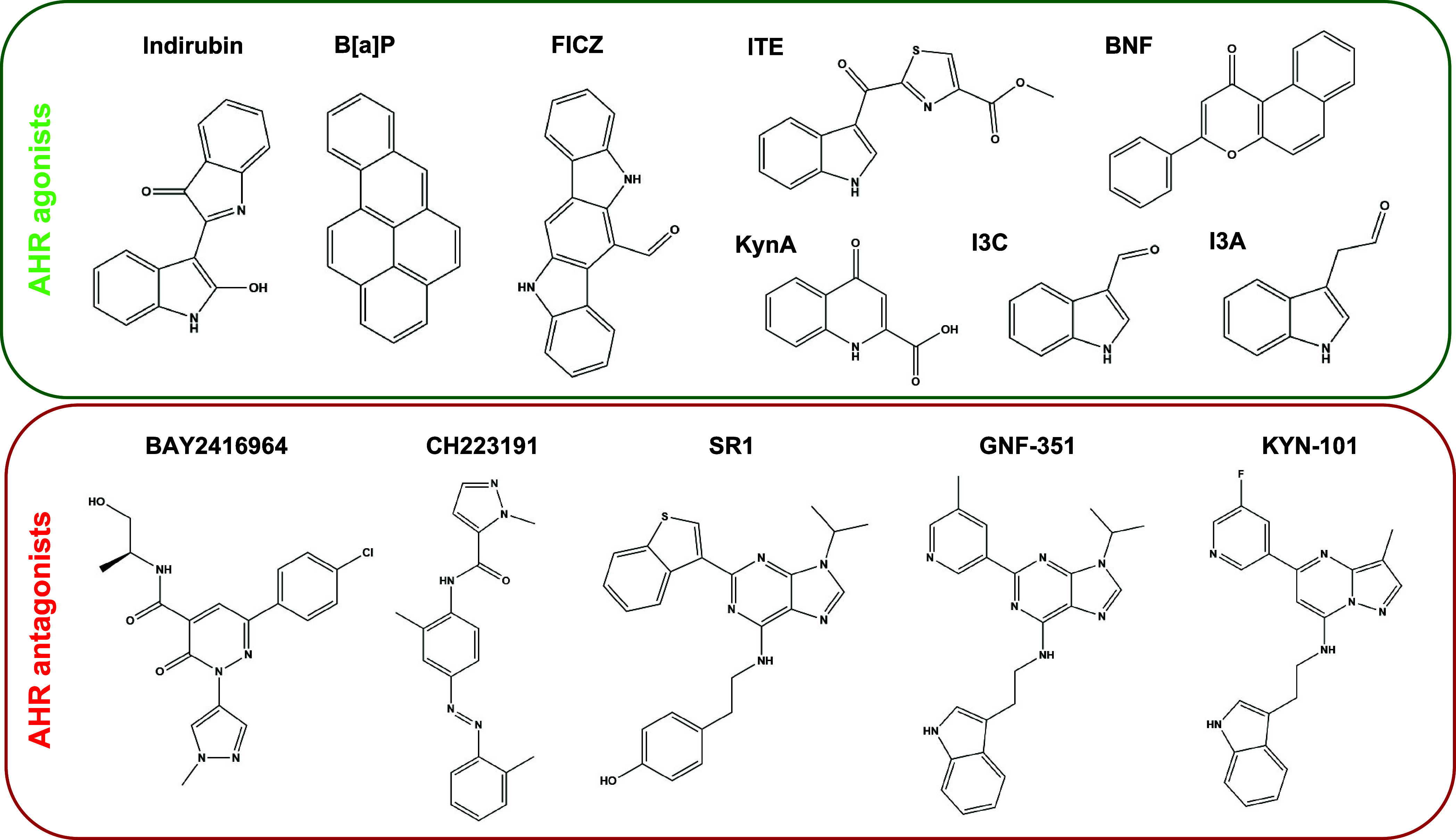
Chemical structures of selected AHR agonists (top) and antagonists (bottom). AHR agonists: Indirubin; B­[*a*]­P, benzo­[*a*]­pyrene; FICZ, 6-formylindolo­[3,2-*b*]­carbazole; ITE, methyl-2-(1H-indole-3-carbonyl)­thiazole-4-carboxylate; BNF, β-naphthoflavone; KynA, kynurenic acid; I3C, indole-3-carboxaldehyde; I3A, indole-3-acetaldehyde. AHR antagonists: BAY2416964; CH223191; SR1, StemRegenin1; GNF-351; and KYN-101.

### 3.1 AHR Agonists Bind within the LBP of the AHR

To investigate binding mechanisms and interaction landscapes of AHR ligands with the AHR complex, we utilized the docking algorithm AC, which can be used for blind docking across the entire surface of the cytosolic AHR protein complex, thanks to its robust force-field-based scoring function and comprehensive sampling approach.^56,57^

Using the structural model of the indirubin-bound AHR complex in conjunction with AC, we successfully reproduced the binding of indirubin within the LBP of AHR (Figure 2A). The predicted binding mode of indirubin closely matched its conformation in the ligand-bound cryo-EM structure of the cytosolic AHR complex (PDB ID: 7ZUB),^52^ as well as the recently published X-ray crystallographic structure of the AHR-ARNT complex (PDB ID: 8XSB),^55^ as indicated by the RMSD values (Figure 2B). Two hydrogen bonds with SER365 and GLN383 were observed (Figure 2C), as previously reported.^52^ We further employed AC to dock B­[*a*]P to the AHR complex, predicting binding of B­[*a*]P within the LBP. The conformation of B­[*a*]P with the lowest score (see Methods) aligned with one of the two binding modes of B­[*a*]­P, previously described in the cryo-EM structure of the B­[*a*]­P-bound AHR complex (Figure S1A).^53^ Additionally, AC-predicted another B­[*a*]P conformation matching the alternative binding mode of B­[*a*]P in the same structure, as well as the binding mode of B­[*a*]P within the AHR LBP of the X-ray crystallographic structure of the B­[*a*]­P-bound AHR-ARNT complex (Figure S1A).^55^ The observed congruence between the experimental and computational data supported the reliability of AC as a suitable tool for the computational assessment of AHR ligand binding.

**Figure 2:**
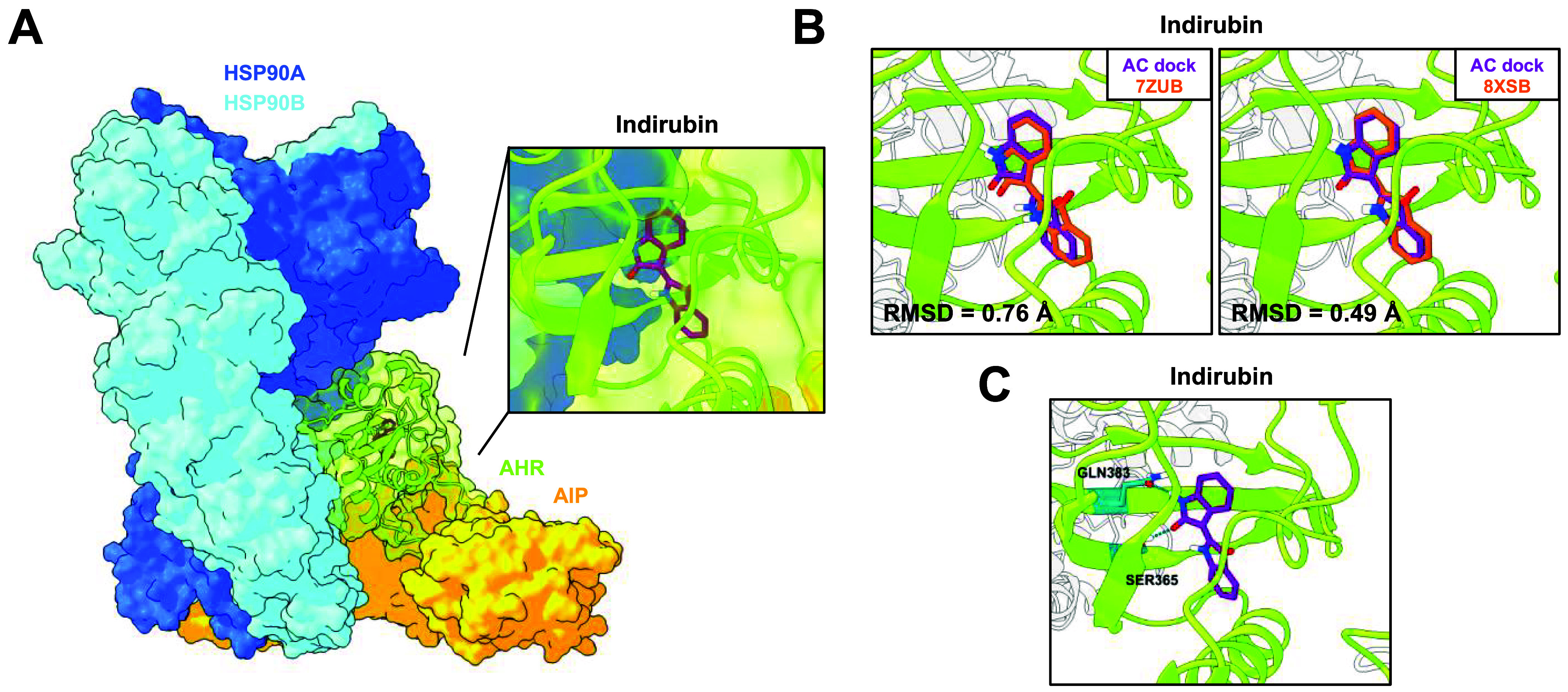
Reproduction of known agonist-binding modes within the LBP of the AHR. (A) Docking was performed based on the cryo-EM structure of the indirubin-bound cytosolic AHR complex, including the AHR, two HSP90 molecules, and one AIP molecule, using the docking algorithm AC. The best conformation of indirubin within the LBP of the AHR, chosen based on the docking score, is shown. (B) The AC-predicted conformation of indirubin overlapped with the conformations of the corresponding ligand, experimentally determined by cryo-EM (PDB ID: 7ZUB) or X-ray crystallography (PDB ID: 8XSB). The RMSD value between the docked and experimentally resolved ligand conformation is given. (C) Prediction of hydrogen bonds for the best conformation of indirubin within the LBP of AHR. The residues contributing to the hydrogen bond formation are colored in turquoise and labeled accordingly. The HSP90 molecules and AIP were colored light gray for a better appreciation of the conformation.

Aiming for a better understanding of the binding mechanism of AHR ligands, we also performed docking studies with other established AHR agonists: BNF, FICZ, ITE, KynA, I3A, and I3C (Figure 1). These molecules are relatively small, planar, and aromatic, chemical properties essential for AHR activation, as previously suggested.^53^ For all AHR agonists, our docking study predicted binding within the LBP of AHR (Figure 3A). KynA is predicted to bind to the LBP only with a protonated carboxylate group and a neutral total charge. This is in agreement with recent findings, suggesting that the LBP is unfavorable for charged ligands due to the predominantly uncharged nature of the residues lining the cavity, imposing a high energy penalty on the desolvation of charged ligands.^52,85^ Similar to indirubin and B­[*a*]­P, AC-predicted conformations of BNF and FICZ aligned with the experimentally resolved conformations of the corresponding ligands of the AHR-ARNT complex (Figure S1A).^55^ To examine potential agonist interactions, we focused on the roles of hydrogen bonds as well as vdW contacts. Besides SER365 and GLN383, three other residues of the LBP (GLY321, SER336, and SER346) were involved in stabilization of the agonist conformations through hydrogen bond formation (Figure 3A,B). Further, vdW contacts were consistently formed across the predicted conformations, involving multiple residues within the LBP of AHR that additionally stabilized the predicted agonist conformations (Figure 3C,D). Interestingly, the conformations of all of the AHR agonists were predicted to be positioned on the same plane in the LBP (Figure S1B), in accordance with recent evidence.^55^ We obtained similar results when docking the agonists to the structure of the AHR complex bound to B­[*a*]P (data not shown).^53^ These findings do not exclude that each agonist engages active-site residues within the AHR LBP to different extents, which may result in differential responses, as previously demonstrated in functional assays, in which distinct agonists (BNF, FICZ, indirubin, ITE) generated varying levels of AHR activation in luciferase assays.^86^

**Figure 3:**
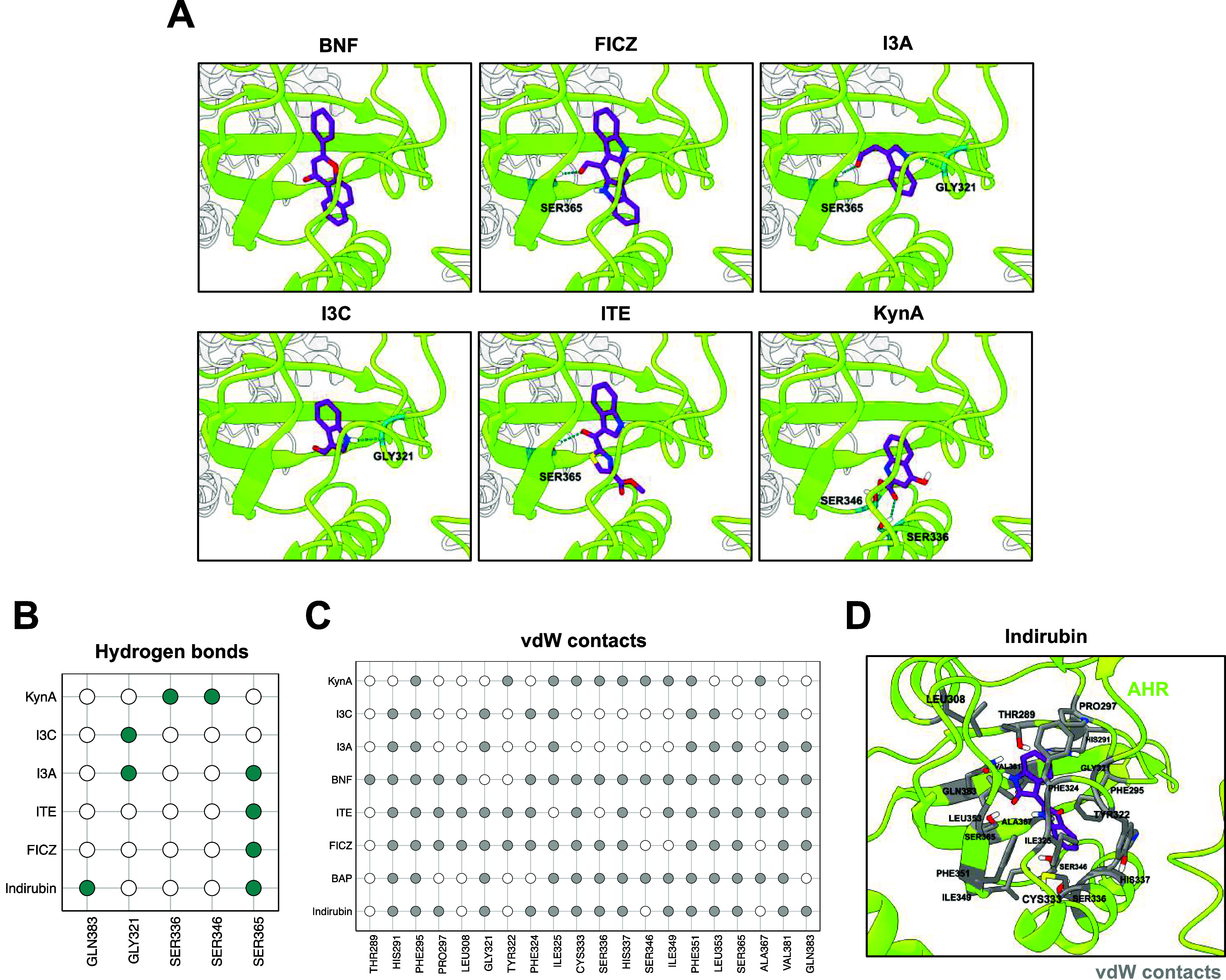
AC predicts binding of AHR agonists within the LBP of the AHR. (A) Docking analysis was performed using the docking algorithm AC and the cryo-EM structure of the indirubin-bound cytosolic AHR complex. The best conformations of AHR agonists in the LBP of AHR are shown with the hydrogen bonds that can be established in the pocket. The residues that contribute to the formation of the hydrogen bonds are colored in turquoise and labeled accordingly. (B) The color (turquoise) in the binary heatmap represents the formation of a hydrogen bond between a residue of the AHR and an AHR agonist. The absence of an agonist indicates that no hydrogen bonds are formed. (C) The best AC-predicted conformations of the AHR agonists were further analyzed for their potential establishment of vdW contacts to residues of the AHR. The color (gray) in the binary heatmap represents the formation of a vdW contact between a residue of the AHR and an AHR agonist. (D) Representative visualization of indirubin (purple) in AHR‘s LBP and labeled AHR residues contributing to the formation of vdW contacts based on (C).

Vina, as one of the most extensively used docking tools in structure-based drug discovery, was used for comparative docking analysis. In alignment with AC, docking with Vina predicted that all agonists fit well into AHR’s LBP (Figure S2).

Overall, our findings support the suitability of the indirubin-bound human HSP90-AIP-AHR cytosolic complex as a model for computational ligand docking studies.

### 3.2 Structural Rearrangement of the Dα-Eα-Loop of AHR Might Be Essential for Binding of AHR Antagonists within the LBP of AHR

Efforts have been made to investigate AHR agonist-binding modes and mechanisms, but the corresponding understanding of AHR antagonists remains comparatively limited. In this study, we strived to explore binding modes and molecular mechanisms of AHR antagonists, including BAY2416964, KYN-101, GNF-351, SR1, and CH223191 (Figure 1).

Surprisingly, the global docking approach with AC did not predict the binding of any of the AHR antagonists within the LBP of AHR. Moreover, Vina also did not predict binding of BAY2416964, GNF-351, or SR1 inside the LBP (Figure 4). Only for KYN-101 and CH223191, potential binding modes were found inside the LBP (Figure 4).

**Figure 4:**
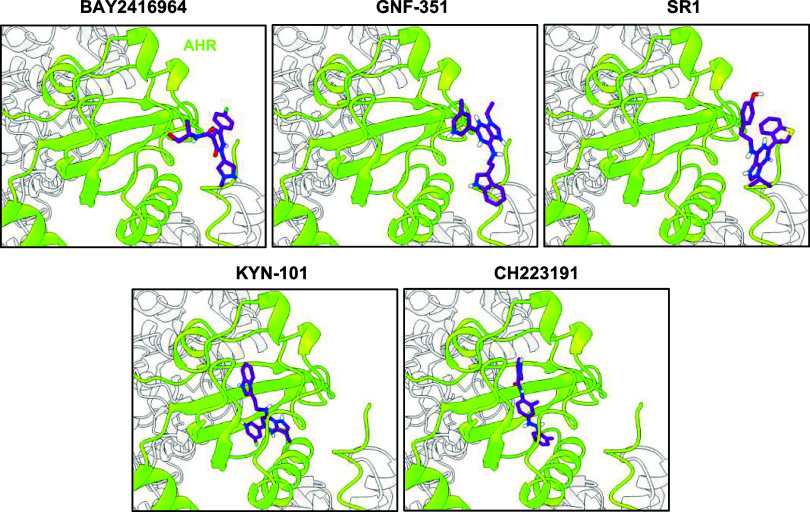
Local docking of AHR antagonists within the LBP of AHR using the docking algorithm Vina. (A) Vina was used to perform local docking of AHR antagonists (BAY2416964, GNF-351, SR1, KYN-101, and CH223191) within the LBP of the AHR. The best conformations of all AHR antagonists in the LBP of the AHR, predicted by Vina and based on their docking scores, are visualized. The HSP90 molecules and AIP were colored light gray for a better appreciation of the conformations.

To investigate whether this finding was due to the rigid protein environment, we performed additional docking calculations with a flexible protein environment. This allowed us to observe different binding modes of AHR antagonists within the LBP (Figure 5). The predicted binding modes of BAY2416964, GNF-351, and SR1 induced a large structural rearrangement of the Dα-Eα-loop of the AHR (Figure 5), while KYN-101 and CH223191 provoked only smaller structural changes.

**Figure 5:**
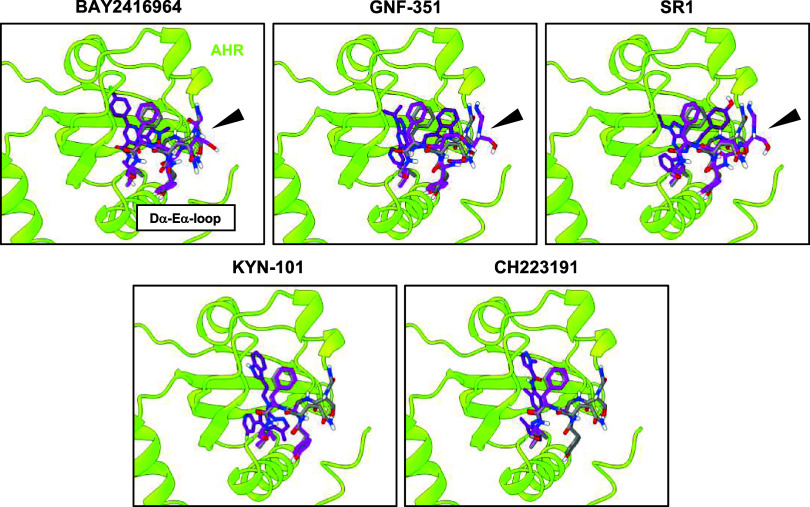
Flexible docking using AC predicts that structural rearrangements of the AHR are required for AHR antagonists to bind within the LBP of the AHR. Docking analysis with the cryo-EM structure of the AHR complex and the AHR antagonists BAY2416964, GNF-351, SR1, KYN-101, and CH223191 was performed using the docking algorithm AC. Throughout the docking calculations, selected residues of the LBP of the AHR were kept flexible. Representative conformations of the antagonists (purple) in the AHR‘s LBP, selected based on the docking score or induction of a structural rearrangement in the Dα-Eα-loop of the AHR (colored in magenta and highlighted with a black arrowhead), are shown. The corresponding residues of the ligand-unbound model are colored in gray.

Our findings suggest that BAY2416964 binding in the LBP could induce conformational changes in the Dα-Eα-loop of the AHR PAS-B domain, potentially a common mechanism required to accommodate the binding of AHR inhibitors. In accordance, a previous report highlighted the potential role of residues 307–329, contributing to the formation of the Dα-Eα-loop, in accommodating GNF-351 in the LBP of AHR.^51^ In addition, a conformational change of this loop has been observed upon ligand binding in very recent structural insights gained from comparison of unbound and bound states of the AHR complex.^53,55^ We hypothesize that this conformational change, most likely supported through the flexibility introduced by two glycines (GLY319, GLY321) within this region, could exert an effect on the interaction landscape of AHR’s C-terminal region with its PAS-B domain—ultimately affecting the stability and integrity of the AHR complex,^51^ or formation of the AHR-ARNT heterodimer. Given the proximity of the Dα-Eα-loop to regions critical for cochaperone interactions and subsequent nuclear translocation, antagonist-induced structural changes within this loop could influence the overall conformational dynamics and functional state transitions of the AHR complex.

### 3.3 AHR Antagonists are Predicted to Bind to Two Sites Distinct from the LBP

The global docking with AC additionally suggested that AHR antagonists may bind to two distinct cavities, hereinafter termed site B and site C (Figure 6). Site B is positioned at the interface of AHR and AIP, whereas site C is localized between the two HSP90 molecules and the PAS-A/PAS-B-linker of the AHR (Figure 6A). For most of the AHR antagonists, several binding modes with similar scores were predicted by AC at the two sites (Figure 6B,C).

**Figure 6:**
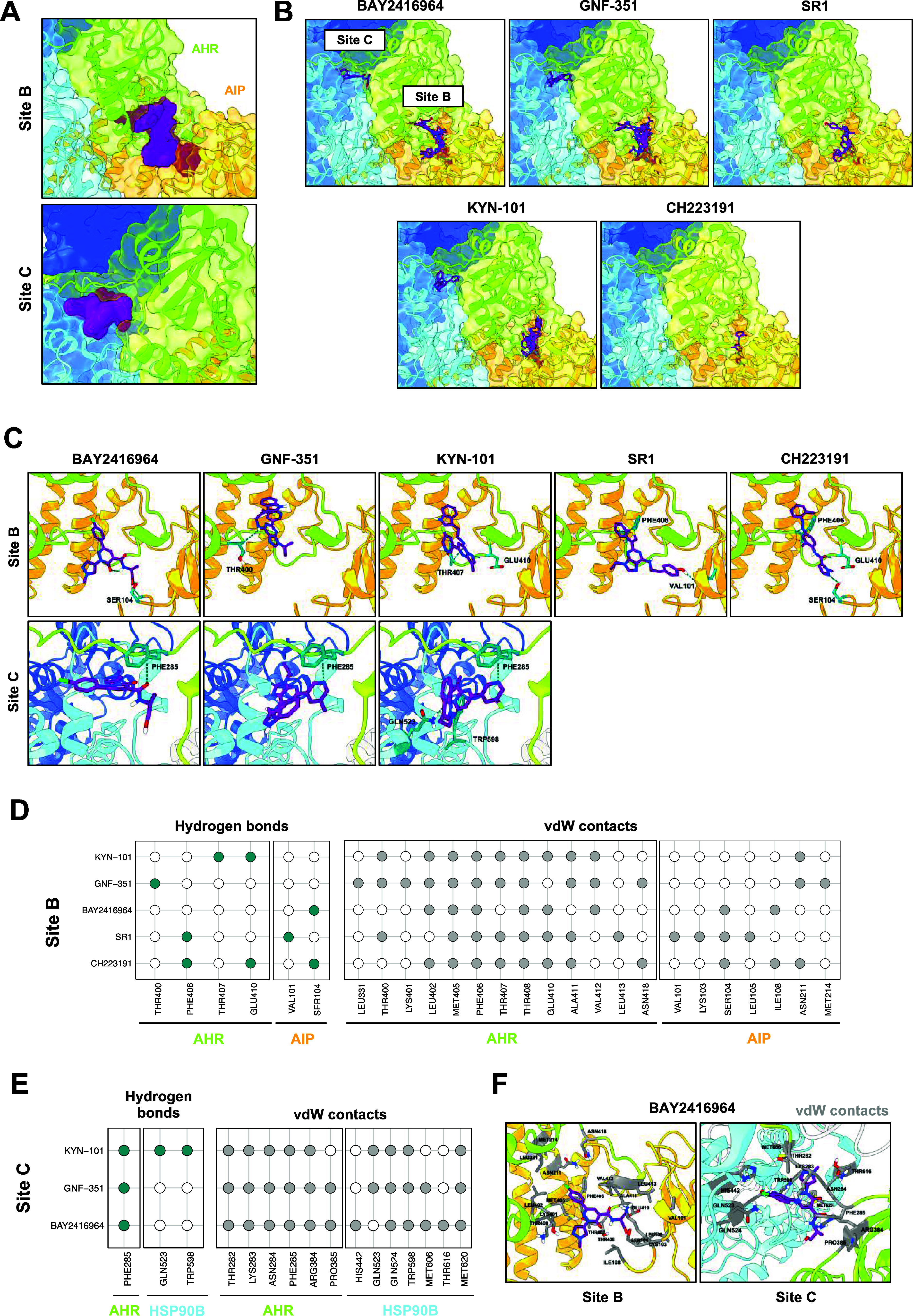
Docking with AC predicts the binding of AHR antagonists outside of the LBP of the AHR. (A) The AHR antagonists BAY2416964, GNF-351, SR1, KYN-101, and CH223191 were docked onto the cryo-EM structure of the AHR complex using the docking algorithm AC. AC-predicted binding of the antagonists in two sites, site B and site C, localized outside of the LBP of the AHR. The surface of all predicted conformations in each site is shown. (B) All predicted binding modes of each AHR antagonist within site B or C. (C) The best AC-predicted conformations of the AHR antagonists within sites B and C, based on their docking scores, are shown with the hydrogen bonds that can be established within the sites. The residues that contribute to the formation of the hydrogen bonds are colored in turquoise and labeled accordingly. (D) The colors (turquoise, hydrogen bond; gray, vdW contact) in the binary heatmap represent the formation of a hydrogen bond or a vdW contact between a residue of AHR or AIP and an AHR antagonist within site B, respectively. (E) Similarly, hydrogen bond formation (turquoise) and establishment of vdW contacts (gray) were analyzed for the best AC-predicted conformations of the AHR antagonists within site C. (F) A representative visualization of BAY2416964 within site B and site C of the AHR complex, with all residues that are involved in vdW contacts, according to (D) and (E), is shown.

For site B, analysis of the predicted binding modes revealed distinct binding patterns driven by the chemical properties of each antagonist. Focusing on the best conformations of each antagonist, different residues, including THR400, PHE406, THR407, and GLU410—belonging to the C-terminal region of AHR—and VAL101 and SER104 of AIP contributed to hydrogen bonding with the antagonists, highlighting their potential importance for binding (Figure 6D). vdW contacts involved residues MET405, PHE406, and THR408 of AHR across different conformations (Figure 6D). Site C was occupied by three of the five antagonists: BAY2416964, KYN-101, and GNF-351 (Figure 6E). In all three cases, AHR PHE285 stabilized its conformation through hydrogen bonding (Figure 6E). The binding modes of these antagonists within the site were similar, leading to the identification of several residues potentially involved in vdW contacts. These included GLN524, TRP598, and MET620 of HSP90B, as well as THR282, LYS283, ASN284, PHE285, and ARG384 of AHR (Figure 6E,F).

We also employed Vina to assess whether we can predict binding modes of the AHR antagonists in both site B and site C, for which we defined new search spaces to perform local docking. With a focus on the best conformations in each site across the different antagonists, based on the respective docking score, the results imply a potential for AHR antagonists to bind to the two sites outside the LBP of AHR (Figure S3).

In conclusion, based on the findings of our docking study, we hypothesize that AHR antagonists may not only bind to the LBP of AHR following structural rearrangements, but also bind to and engage with two previously uncharacterized cavities within the cytosolic AHR complex. In line, previous evidence suggests that regions outside the LBP could contribute to AHR activation.^86^ Binding of antagonists to the novel sites could potentially stabilize interactions between the AHR and the cochaperones of the complex, thereby maintaining its integrity, or influence agonist binding to AHR’s LBP. Until now, the prevailing hypothesis has been that AHR antagonists act by competing with agonists in the LBP. Indeed, multiple competitive ligand-binding assays have been conducted using AHR agonists and increasing concentrations of AHR antagonists, including GNF-351, SR1, and CH223191, demonstrating that AHR antagonists compete with agonists for binding to the LBP.^87–89^ However, these studies do not rule out the possibility of allosteric mechanisms of the antagonists—an idea that had been proposed earlier.^90^ There is increasing evidence that suggests allosteric antagonism of the AHR through compounds and drugs.^91–94^ For instance, instead of displacing a radiolabeled agonist from the LBP of AHR, carvones bind allosterically to AHR, inhibiting heterodimerization of AHR with its nuclear interaction partner, ARNT.^92^ Similarly, the cyclopentanone jasmone has been classified as a potent allosteric antagonist of AHR, affecting AHR-ARNT heterodimerization.^93^ Therefore, it is conceivable that allosteric regulation could occur at the level of the cytoplasmic AHR complex, an aspect that is yet to be explored.

### 3.4 Pocket Prediction Tool Fpocket Supports the Possibility of Ligand Binding Outside of the LBP of AHR

To corroborate our hypothesis on potential AHR antagonist binding outside of the LBP of AHR, we utilized Fpocket, a protein pocket detection tool, to examine the cryo-EM structure of the indirubin-bound AHR complex. Prediction of protein pockets by Fpocket is based on Voronoi tessellation and α spheres with application of a simple scoring function to rank the predicted pockets.^60^ Beyond ligand-binding site identification, Fpocket also provides a druggability score, which evaluates the likelihood of each identified pocket to bind a drug-like molecule.^60,95^

Fpocket identified pockets representing the ATP-binding sites of the HSP90 molecules (Figure 7A, **ATP**) and the LBP of AHR (Figure 7A,B), respectively. With a druggability score of 0.96, the LBP was the site with the highest predicted likelihood of binding to drug-like molecules. Strikingly, Fpocket additionally predicted two pockets that corresponded to the AC-predicted binding sites (sites B and C) of the AHR antagonists (Figure 7A). Site B was identified as an elongated cavity located at the interface of the AHR LBP and the AIP (Figure 7A,B). Analysis with Fpocket revealed that site B comprised two closely positioned subpockets with druggability scores of 0.54 and 0.29, respectively, suggesting potential binding of a drug-like molecule. Similarly, site C was characterized as another long cavity, located close to the AHR PAS-A/PAS-B-linker, positioning itself at the interface of the HSP90 molecules (Figure 7A,B). With a druggability score of 0.81, this site represents a cavity with a high likelihood of binding pharmacological compounds.

**Figure 7:**
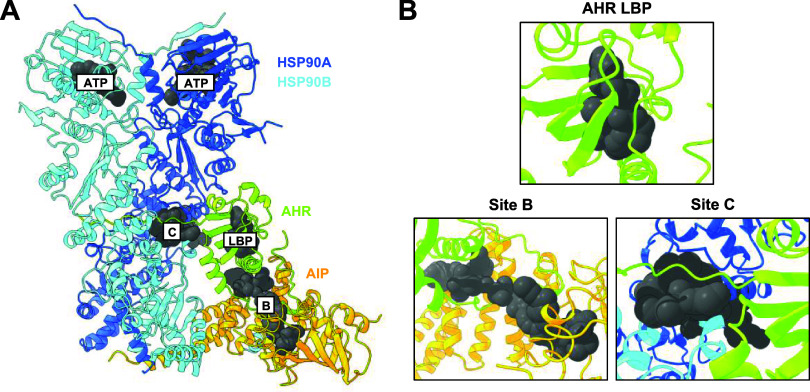
Prediction of binding pockets within the cryo-EM structure of the AHR complex corresponding to sites B and C. (A) Binding pockets were predicted using Fpocket on the cryo-EM structure of the AHR complex. The LBP within the AHR and the two alternative binding sites outside the LBP are labeled LBP, B, and C, respectively. Predicted ATP-binding sites within HSP90 are also indicated. (B) Close-up view of the predicted pockets. The LBP is located within the AHR, site B interacts with the AHR and AIP, and site C is positioned between the AHR and the two HSP90 molecules.

Overall, the use of the protein pocket detection tool Fpocket supports our hypothesis that therapeutically relevant small molecules may bind to alternative binding sites within the cytosolic AHR complex. Notably, the alternative binding sites were larger and chemically more permissive than the LBP, suggesting a potential for more diverse ligand engagement.

### 3.5 MD Simulations Reveal the Most Stable Binding of BAY2416964 in the LBP and Site C

To capture the dynamic behavior and conformational changes of the system beyond what static structures reveal, we performed MD simulations of the ligand-free and ligand-bound AHR complex. For a better understanding of AHR agonism and antagonism, we decided to focus on indirubin as a well-described AHR agonist with a known binding mode in the LBP, and on BAY2416964 as a key AHR antagonist with clinical application.

Clustering analysis of ligand conformations across the MD trajectories showed that indirubin remained very stable in the LBP of AHR (proportion first cluster = 83%, *N* = 8 clusters) (Figure 8A), showing an average RMSD of 0.32 nm (Figure 8F), and aligning with previous studies.^52^ Within the LBP, BAY2416964 showed an adequate stability (1st cluster proportion = 64%, *N* = 14 clusters) (Figure 8B). The first three clusters showed structural differences related to loop regions, particularly the loop connecting Dα to Eα (Figure 8C). In site B, BAY2416964 adopted a wide range of binding conformations (1st cluster proportion = 48%, *N* = 62 clusters) (Figure 8D). By contrast, BAY2416964 showed the greatest conformational stability in Site C (1st cluster proportion = 85%, *N* = 6 clusters) (Figure 8E). A similar trend was reflected in the RMSD of the ligands with respect to their initial positions (Figure 8F).

**Figure 8:**
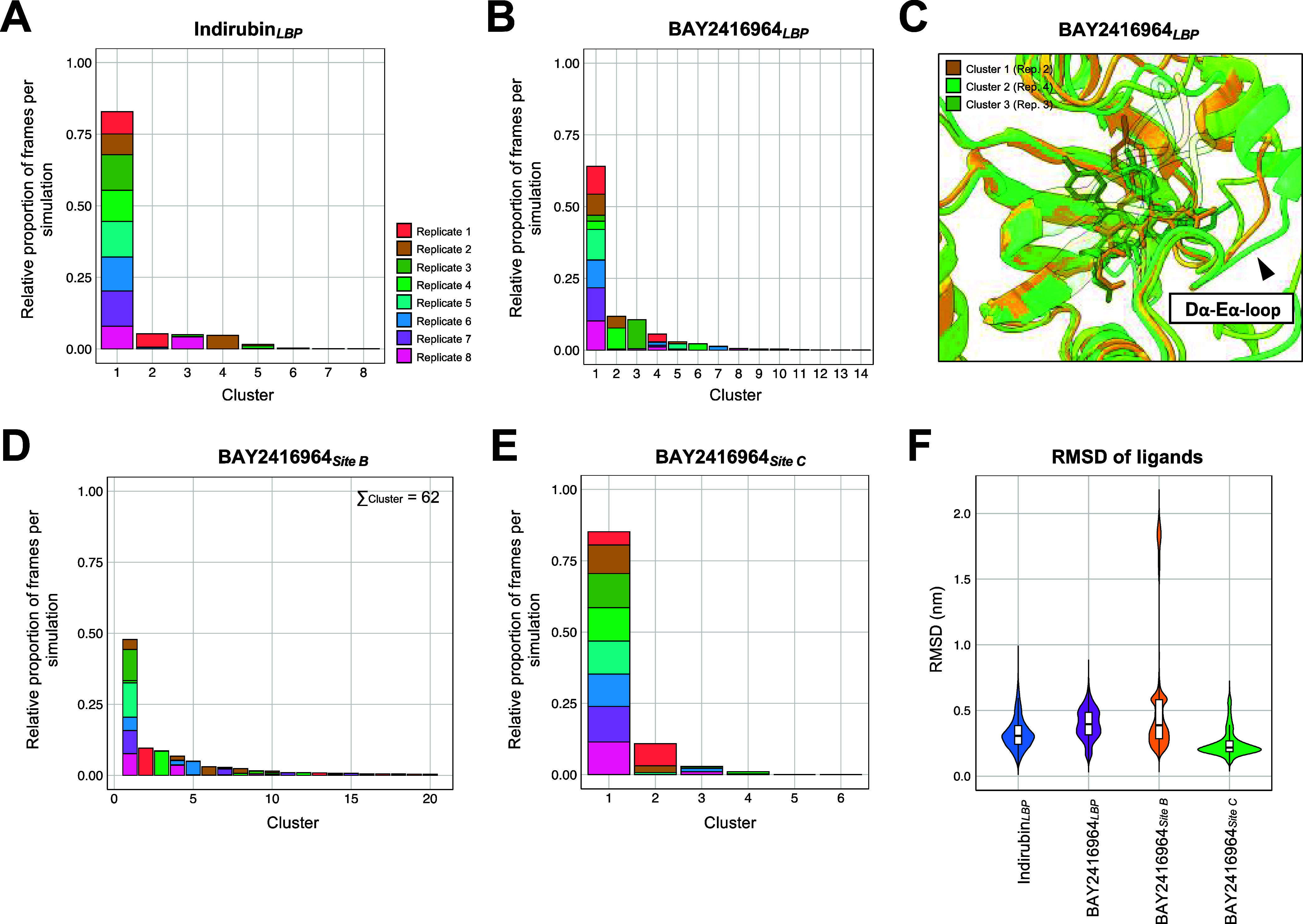
Clustering and RMSD analyses based on MD simulations support binding of BAY2416964 in the LBP and sites B and C. (A) Clustering of conformational states of indirubin in the LBP across the replicates (*N* = 8 per MD system). The proportion of replicates per cluster is shown. (B) Clustering analysis of BAY2416964 in the LBP. (C) Superimposition of the central structures of clusters 1–3, deriving from replicate 2, 4, and 3, respectively, for BAY2416964 in the LBP. Structures are colored based on the color of the replicate of origin in (B), with BAY2416964 shown in darker shades. Structural differences in the Dα-Eα-loop are indicated by a black arrowhead. (D) Clustering analysis of BAY2416964 in site B. Only the first 20 of all 62 clusters are shown. (E) Clustering analysis of BAY2416964 in site C. (F) Violin plots showing RMSDs across all replicates for indirubin in LBP and BAY2416964 in LBP, site B, and site C (*N* = 8 per MD system).

To investigate the ligand–protein interactions in the different sites, we calculated per-residue vdW and electrostatic interaction energies, as well as hydrogen bonds. In agreement with the findings of Gruszczyk et al.,^52^ our MD simulations showed involvement of SER365 and GLN383 contributing to stabilization of indirubin in the LBP through electrostatic interactions and hydrogen bond formation (Figure 9A). In addition, we identified HIS291, PHE295, and PHE351 as the main contributors to vdW interactions. These residues had previously been identified as part of the residues that play a key role in accommodating indirubin’s planar structure within the site.^52^

**Figure 9:**
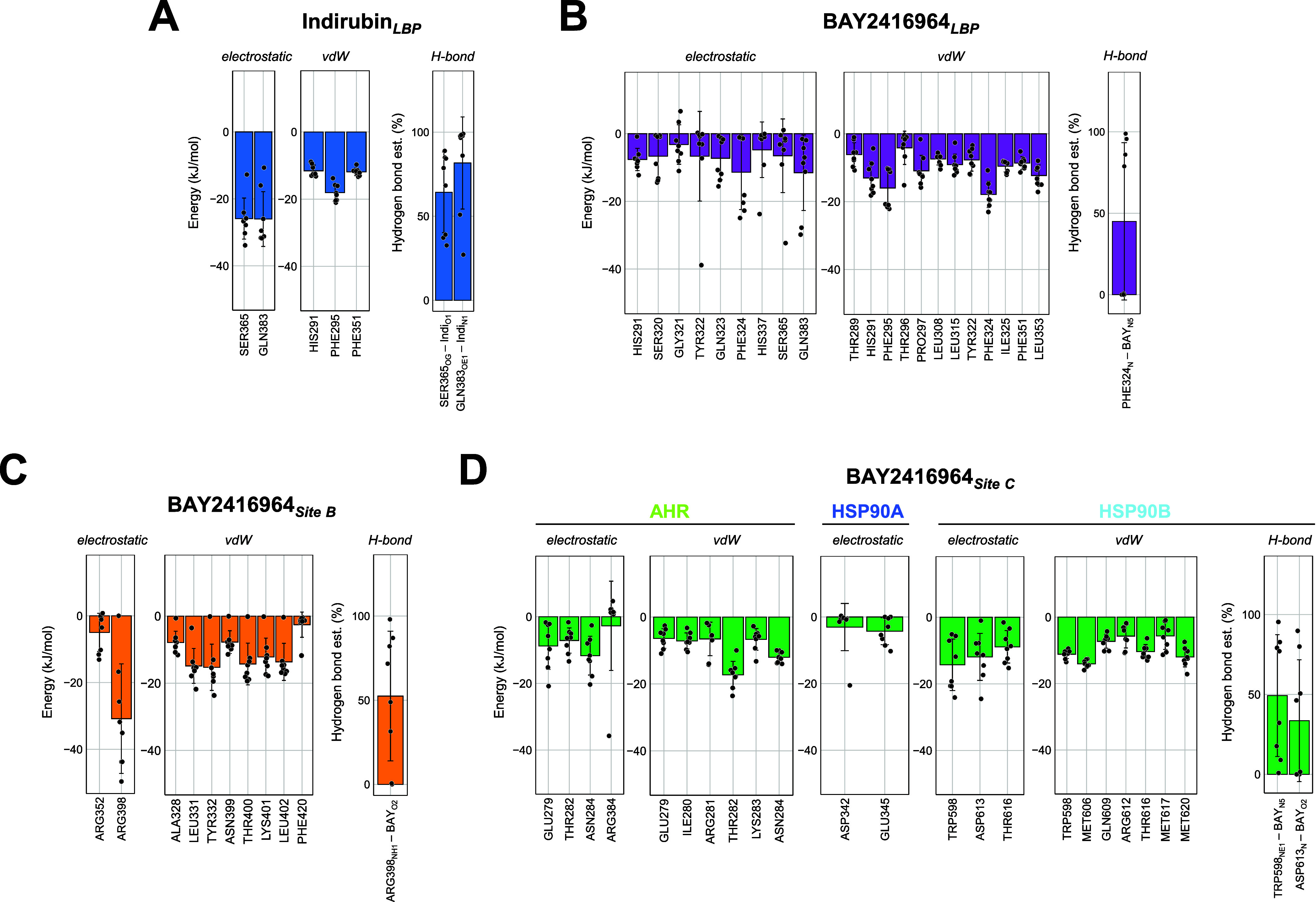
Analyses of nonbonded interaction energies and hydrogen bonds reveal residues important for the binding of indirubin and BAY2416964 to the cytosolic AHR complex. Nonbonded interaction energies (electrostatic and vdW contacts) and hydrogen bond formation between selected contact residues (≤0.42 nm distance for ≥30% of the simulation time) were calculated across MD simulation replicates. Bar graphs show the mean values ± SD across replicates (*N* = 8 per MD system), with individual data points representing replicate means. For clarity, only residues with an interaction energy of ≤−10 kJ/mol in at least one replicate, or a hydrogen bond frequency of ≥30% are shown. (A) Indirubin in the LBP (Indirubin_LBP_), (B) BAY2416964 in the LBP (BAY2416964_LBP_), (C) BAY2416964 in site B (BAY2416964_Site B_), and (D) BAY2416964 in site C (BAY2416964_Site C_).

PHE324 was the main contributor to the stabilization of BAY2416964 in the LBP, both by vdW interactions and by hydrogen bond formation (Figure 9B). BAY2416964 also interacted with multiple AHR residues within the LBP, some of which were unique to the interaction of the antagonist with this site. These included SER320, TYR322, and GLN323 through electrostatic interactions and THR289, THR296, LEU308, and LEU315 through vdW contacts (Figure 9B). However, some of the strongest interactions were established with GLN383 (electrostatic) and HIS291, PHE295, and LEU353 (vdW). High data variance may be attributed to the lower conformational stability of the antagonist in the LBP, compared to indirubin.

Interaction energy analysis of BAY2416964 in site B revealed that it was primarily interacting with residues of the AHR. Particularly, ARG398 highly contributed to stabilization of the antagonist in this site through hydrogen bonding and formation of electrostatic interactions (Figure 9C). Several residues contributed via vdW contacts, such as LEU331, TYR332, and residues of the C-terminal loop, including THR400, LYS401, and LEU402 (Figure 9C). Notably, in replicate one, these residues did not contribute to the stabilization of BAY2416964 due to its extensive exploration of the site, which is in alignment with the higher variability in the conformational states of BAY2416964 in site B (Figure 8D/F). Nevertheless, no unbinding event occurred. None of the AIP residues were identified as contact residues of BAY2416964 in site B.

In site C, BAY2416964 primarily interacted with AHR and HSP90B, while HSP90A played only a minor role (Figure 9D). GLU279, THR282, and ASN284 of AHR were similarly involved in electrostatic interactions, with THR282 also exhibiting the strongest vdW interaction. The high variance observed for ARG384 was due to its involvement in ligand interaction in only one of the replicates (Figure 9D). TRP598, ASP613, and THR616 of HSP90B stabilized the antagonist in site C through electrostatic interactions, with TRP598 and ASP613 additionally forming hydrogen bonds (Figure 9D). Further stabilizing vdW interactions were contributed by TRP598, MET606, THR616, and MET620 of HSP90B. The large variances in interaction energies and hydrogen bonding reflect the sampling of different conformations across the replicates.

Taken together, our MD simulations support stable binding of BAY2416964 within the LBP of AHR and even more so in site C. The less stable binding of BAY2416964 in site B suggests a more flexible mode of allosteric binding of the antagonist at this site.

### 3.6 Backbone Stability and Residue Fluctuations of AHR in the Different Ligand-Free and Ligand-Bound Systems

To explore the conformational stability and structural fluctuations of the AHR across the ligand-free and ligand-bound systems, we analyzed the backbone RMSD and RMSF of the AHR, including the different structural regions that constitute the LBP (Figure 10A). A comparison between the apo- and indirubin-bound systems revealed a slight decrease in the mean RMSD and RMSF value within the Cα-Dα region upon ligand binding (Figure 10B,C, **label 1**). The reduction of the RMSF in the Cα-Dα region observed for the indirubin-bound system is in agreement with the literature.^52^

**Figure 10:**
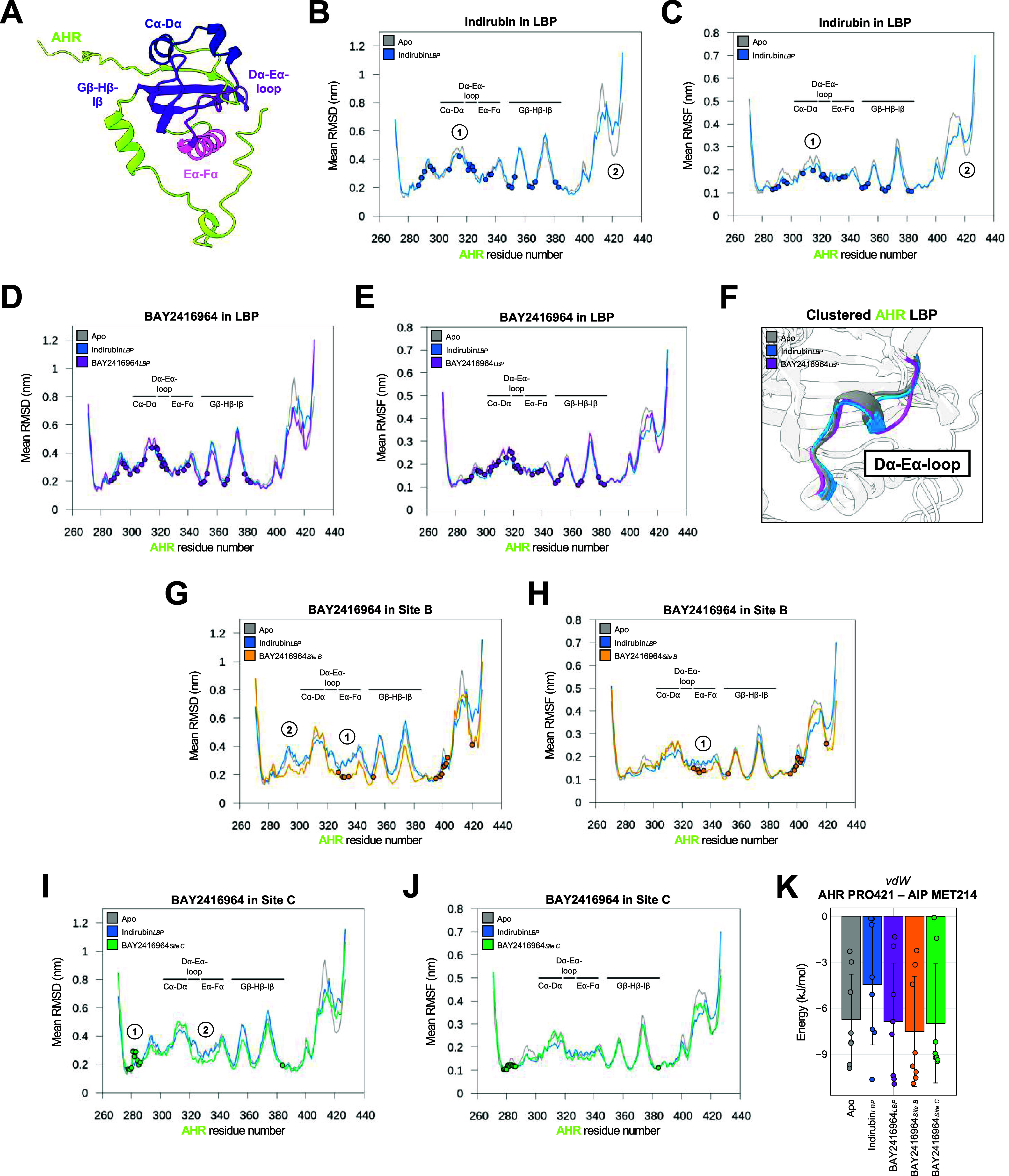
Ligand binding to different sites leads to distinct RMSD and RMSF profiles of the AHR backbone. (A) AHR with selected secondary structures forming the LBP. (B) Mean per-residue backbone RMSD of AHR for Apo and Indirubin bound to the LBP (Indirubin_LBP_) systems (*N* = 8 per MD system), showing backbone stability. Structural regions from (A) are annotated; numbered circles mark differences; dots indicate indirubin contact residues (≤0.42 nm for ≥30% of simulation). (C) As in (B), but showing per-residue RMSF. (D, E) Same analyses for BAY2416964 bound in the LBP. (F) Clustered LBP conformations of Apo, Indirubin_LBP_, and BAY2416964_LBP_ systems, highlighting Dα-Eα loop differences. (G, H) RMSD and RMSF for BAY2416964 bound to site B. (I, J) RMSD and RMSF for BAY2416964 bound to site C. (K) Nonbonded (vdW) interaction energies between AHR PRO421 and AIP MET214, shown as mean ± SD (*N* = 8 per MD system) with individual points representing the mean value from each replicate.

Interestingly, binding of BAY2416964 within the LBP resulted in RMSD and RMSF values comparable to those of the apo system, indicating no major changes in structure and flexibility of the AHR upon binding of the antagonist (Figure 10D,E). However, given our prior findings on the potential relevance of the structural rearrangement of the Dα-Eα-loop induced by BAY2416964 within the LBP, we clustered the AHR PAS-B backbone to explore the conformational states of this region. The clustering study indicated that binding of BAY2416964 within the LBP stabilized a structural conformation of the Dα-Eα-loop distinct from the prevalent conformation in the ligand-free and indirubin-bound systems (Figure 10F).

Binding of BAY2416964 in site B significantly stabilized the AHR in a conformation close to the experimentally resolved one and reduced fluctuations, both in regions proximal to the binding site (Figure 10G–H, **label 1**) and at more distant regions (Figure 10G, **label 2**).

Binding of BAY2416964 in site C resulted in minor changes in the RMSD and the RMSF of the AHR backbone compared with the apo- and indirubin-bound systems. Specifically, the region encompassing the N-terminal linker residues ILE280 to PHE285 showed a slight structural change, probably to accommodate the antagonist in this site (Figure 10I, 1). Further, BAY2416964 in site C notably decreased the RMSD in the Eα-Fα region, which is distant to the binding site, in a manner similar to BAY2416964 in site B; however, less pronounced (Figure 10J, **label 2**). This finding indicates that binding of a hydrophobic molecule, such as BAY2416964, in site B or C could stabilize the AHR conformation within the cytosolic complex.

Upon comparison of all MD simulations, we observed a pronounced increase in the mean RMSD and RMSF around the region near PRO421 only for the indirubin-bound system (Figure 10B, **label 2**). Analysis of the nonbonded interaction energies of PRO421 indicated that its interaction with MET214 of AIP was reduced, particularly in the indirubin-bound system compared to the apo and BAY2416964-bound systems (Figure 10K). This observation may hint at a potential role of this interaction in indirubin-mediated activation of the AHR. We suggest that this may be of interest for future investigation.

In summary, based on the analysis of the backbone RMSD and RMSF of AHR across the different systems, BAY2416964 in sites B and C influenced backbone conformations and residue fluctuations more than BAY2416964 in the LBP. However, BAY2416964 in the LBP induced a conformational change in the Dα-Eα-loop, not observed in the ligand-free or agonist-bound systems.

### 3.7 *In Silico* Alanine Mutation of the AHR Complex Suggests Potential Target Residues for Experimental Validation of the Proposed Allosteric Binding Sites of BAY2416964

Understanding the interactions formed by BAY2416964 in the different potential binding sites of the AHR complex can guide their experimental validation. For this purpose, we subjected all residues of the three different BAY2416964-bound systems (LBP, site B, and site C) to *in silico* mutation to alanine. Focusing on potentially targetable residues based on our interaction energy analysis (see 3.5.), the results suggest that GLN383 (LBP), TYR332, ARG398, THR400, LYS401, and LEU402 (Site B), as well as THR282 and ASN284 of AHR, and ASP613, THR616, and MET620 of HSP90B (Site C) could be mutated without disrupting the AHR complex, while affecting the proposed antagonist binding sites (Figure 11). These residues could therefore serve as candidates for mutational studies aimed at validating our *in silico* findings from the docking and MD simulation analyses.

**Figure 11:**
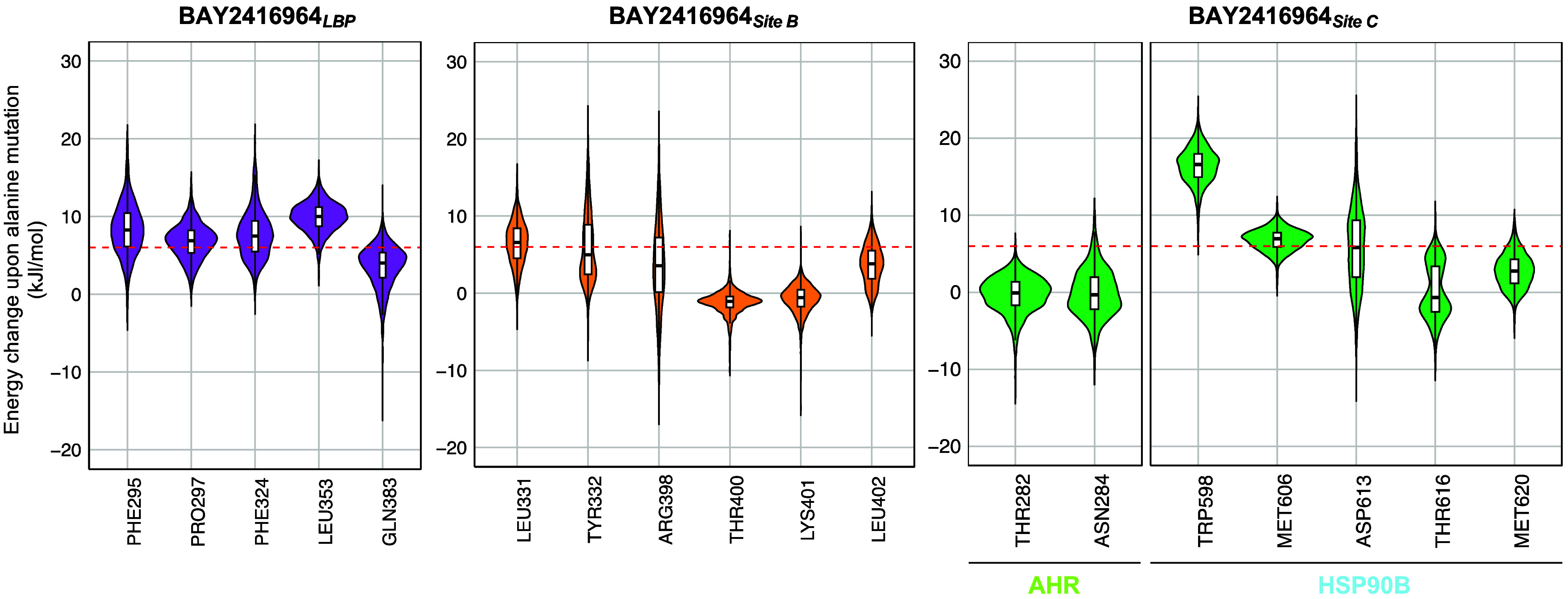
*In silico* alanine mutation of selected BAY2416964 contact residues can inform experimental point mutation studies. For each of the three BAY2416964-bound MD systems: (1) LBP (left), (2) site B (middle), and (3) site C (right), 201 frames were extracted from each replicate (*N* = 8 per MD system). Each residue was subjected to an *in silico* alanine mutation using the selected frames. Violin plots show the resulting Gibbs free energy upon alanine mutation of selected residues. Selected residues were those involved in hydrogen bond formation or nonbonded interactions (averaged value ≤−10 kJ/mol across all replicates) with BAY2416964 in the respective binding site. The dashed red line indicates a 6 kJ/mol threshold below which an amino acid mutation is unlikely to affect the AHR complex structure. Mutations of these amino acids could be used to experimentally test whether this abrogates BAY2416964 binding.

Recent mutational studies established experimental evidence for the importance of SER365 and GLN383 in the interaction of indirubin with the AHR.^52,53^ Given that GLN383 may also contribute to binding of BAY2416964 in the LBP, its mutation is unlikely to allow distinguishing between effects on agonist and antagonist binding. We therefore suggest targeting other residues, such as PRO297 or PHE324, probably more critically involved in the binding of BAY2416964 than indirubin within the LBP. However, it is necessary to exclude that observed effects upon mutation are not attributable to changes in the AHR complex structure. Interestingly, prior research by Soshilov and Denison examined how murine AHR LBP mutagenesis affects the binding and activation by 12 structurally diverse ligands.^96^ The authors identified PHE318 of murine AHR (PHE324 of human AHR) as the residue able to determine agonist/antagonist behavior of AHR ligands, including BNF. Its mutation also decreased HSP90 binding to the murine AHR.^96^ It would therefore be of interest to investigate the impact of the human PHE324 mutation on the inhibitory function of BAY2416964, as well as on agonist binding and the composition of the cytosolic AHR complex.

In contrast, although we propose TYR332 and ARG398 as targets for mutational analysis of site B, a recent study by Diao et al.^55^ demonstrated that mutations of these residues reduce AHR transcriptional activity. Specifically, mutation of ARG398 to glutamic acid resulted in a predominant cytosolic localization of the AHR, suggesting that the residue is probably crucial for protein–protein interactions affecting the nuclear translocation, required for subsequent transactivation of the AHR.^55^ These findings suggest that validating the binding of BAY2416964 to site B may be challenging. However, they also support the hypothesis that BAY2416964 interacts with key residues, including TYR332, ARG398, and residues of the C-terminal loop, that are essential for effective activation of the AHR, ultimately presenting a mechanism by which the antagonist could exert its inhibitory function. Lastly, given that several point mutations in the N-terminal linker of the AHR markedly affect AHR’s interaction with AIP,^52^ mutational studies need to closely monitor the interaction between the complex partners and the stabilization of the complex as a whole when validating the binding of BAY2416964 in site C.

## 4 Conclusions

Altogether, our computational study highlights novel potential binding mechanisms for AHR ligands. While agonists consistently bind the canonical LBP, AHR antagonists may exert their effect through binding to the LBP upon induction of structural rearrangements of the AHR, or through previously unrecognized allosteric sites outside of the LBP. The binding of BAY2416964 in site B was shown to be conformationally flexible, but no unbinding event was observed over the simulated period of 1.6 μs. BAY2416964 in site C, on the other hand, stably interacted with this pocket and was stabilized through residues of the AHR and predominantly one of the HSP90 molecules. These findings may challenge competitive antagonism and expand the view of AHR regulation. Experimental studies are required to validate these predictions and explore their implications for selective AHR modulation in therapeutic settings.

While the findings of our study lay the groundwork for further research, there are several limitations to consider. Molecular docking, though widely used to predict protein–ligand interactions, has inherent constraints related to sampling and scoring. Different docking tools (e.g., AC and Vina) use distinct scoring functions, leading to variable binding pose predictions, and experimental validation is necessary to establish relevant binding modes. Moreover, docking represents a static view of protein–ligand interactions, whereas proteins are inherently dynamic. We addressed these limitations (1) by incorporation of flexibility in key residues using AC and (2) by the performance of MD simulations to better capture conformational dynamics and to assess binding stability. Additional limitations concern the unresolved regions in the indirubin-bound AHR cryo-EM structure and the absence of certain interaction partners, which may limit the accuracy of predicted binding pockets. Although recent crystallographic data on the DNA-bound AHR–ARNT complex suggest a multistep activation mechanism,^55^ they do not clarify how the bHLH/PAS-A domain engages with HSP90 prior to ligand binding. Related to this, the decreased backbone RMSD of AHR upon binding of BAY2416964 in sites B and C may hint at the presence of a hydrophobic factor that is needed to further stabilize the system. Finally, although our study focused on docking and MD simulations, these approaches cannot replace experimental evidence. Rather, our aim is to provide guidance for future experimental studies.

## Supplementary Material



## Data Availability

Processed MD simulation trajectory files, as well as portable binary input and molecular structural files containing information on the starting structures of the simulations, the molecular topologies and all simulation parameters are publicly available on zenodo (10.5281/zenodo.16909114).

## Notes

Prediction and reproduction of known AHR agonist-binding modes within the LBP of AHR by molecular docking with AC (Figure S1); local docking of AHR agonists within the LBP of AHR using the docking algorithm Vina (Figure S2); local docking of AHR antagonists to sites B and C with Vina (Figure S3); backbone RMSD of the AHR complex (excluding AIP residues 2–165) across all MD systems and simulations after superimposition to itself (Figure S4) (PDF)

## Abbreviations

AC: Attracting Cavities
AHR: Aryl hydrocarbon receptor
AIP: AHR-interacting protein
ARNT: AHR nuclear translocator
ATP: Adenosine triphosphate
B­[*a*]­P: Benzo­[*a*]­pyrene
bHLH: Basic helix–loop–helix
BNF: β-naphthoflavone
Cryo-EM: Cryo-Electron microscopy
FACTS: Fast Analytical Continuum Treatment of Solvation
FICZ: 6-formylindolo­[3,2-*b*]­carbazole
HSP90: Heat shock protein 90
I3A: Indole-3-acetaldehyde
I3C: Indole-3-carboxaldehyde
ITE: Methyl-2-(1H-indole-3-carbonyl)­thiazole-4-carboxylate
KynA: Kynurenic acid
LBP: Ligand-binding pocket
MD: Molecular dynamics
MMFF: Merck Molecular Force Field
PAS: PER-ARNT-SIM
PDB: Protein Data Bank
PER: Periodic circadian protein
PTGES3: Prostaglandin E synthase 3
RIC: Random initial conditions
RMSD: Root mean square deviations
RMSF: Root mean square fluctuations
SD: Standard deviation
SIM: Single-minded protein
SR1: StemRegenin1
Vina: AutoDock Vina
vdW: van der Waals
XAP2: X-associated protein 2
