# Supplementary material for: Exploration of Agonist and Antagonist Binding Sites within the Cytosolic AHR Complex Using Molecular Modeling
Source: ACS Omega. 2026 Mar 5;11(10):16070–87. doi: 10.1021/acsomega.5c10598 (PMC13000621; doi:10.1021/acsomega.5c10598)
Supplement: Supplementary file 1 [file ao5c10598_si_001.pdf]

Exploration of Agonist and Antagonist Binding Sites within the Cytosolic AHR Complex Using Molecular Modeling

Ivana Karabogdan[a,b], Francisco Yanqui-Rivera[a,b], Deepak Sayeeram[a,b], Ahmed Sadik[a,b], Aubry K. Miller[c], Saskia Trump[d], Ute F. Röhrig[e,1,], Christiane A. Opitz[a,f,1,*]*

a German Cancer Research Center (DKFZ), Heidelberg, Division of Metabolic Crosstalk in Cancer and the German Cancer Consortium (DKTK), DKFZ Core Center Heidelberg, 69120 Heidelberg, Germany.

b Faculty of Bioscience, Heidelberg University, 69120 Heidelberg, Germany.

c Cancer Drug Development, German Cancer Research Center (DKFZ), 69120 Heidelberg Germany.

d Molecular Epidemiology Unit, Berlin Institute of Health at Charité and the German Cancer Consortium (DKTK), Partner Site Berlin, a partnership between DKFZ and Charité - Universitätsmedizin Berlin, 10117 Berlin, Germany.

e Molecular Modelling Group, SIB Swiss Institute of Bioinformatics, CH-1015 Lausanne, Switzerland.

f Neurology Clinic and National Center for Tumor Diseases, 69120 Heidelberg, Germany.

1 these authors contributed equally, * corresponding authors

Corresponding authors:

Christiane A. Opitz –Email: c.opitz@dkfz-heidelberg.de

Ute F. Röhrig –Email: ute.roehrig@sib.swiss

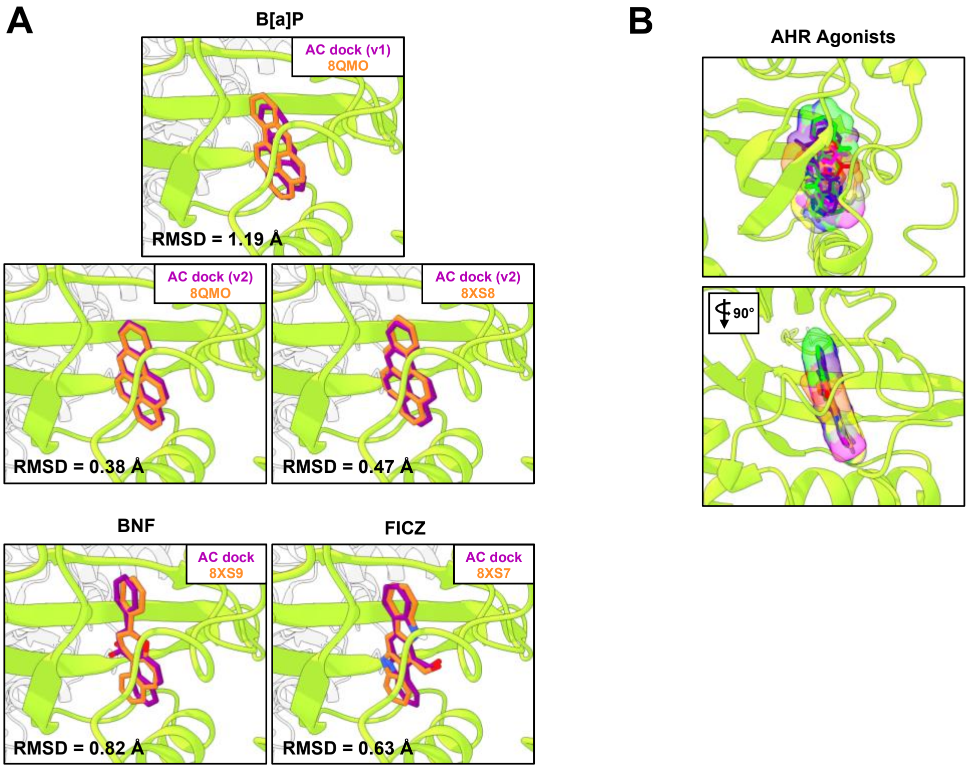

Figure S1. Prediction and reproduction of known AHR agonist binding modes within the LBP of AHR by MD simulation with AC. (A) Two AC-predicted conformations of B[a]P (AC dock v1/v2) superimposed with the conformations of the corresponding ligand experimentally resolved by cryo-EM (PDB ID: 8QMO) and X-ray crystallography (PDB ID: 8XS8). The best pose of B[a]P within the LBP of AHR is represented by (v1). For the cryo-EM structural model (PDB ID: 8QMO), two possible orientations of B[a]P were suggested (see B[a]P; top and lower left panel). The AC-predicted conformations of BNF and FICZ were superimposed with the conformations of the corresponding ligand, experimentally resolved by X-ray crystallography (PDB ID: 8XS7/8XS9). The RMSD value between the docked and experimentally resolved ligand conformation is given. (B) Superimposition of the best conformations of indirubin, B[a]P, BNF, FICZ, I3A, I3C, ITE, and KynA, differentially colored, indicates positioning of the ligands in one plane in the LBP of the AHR.

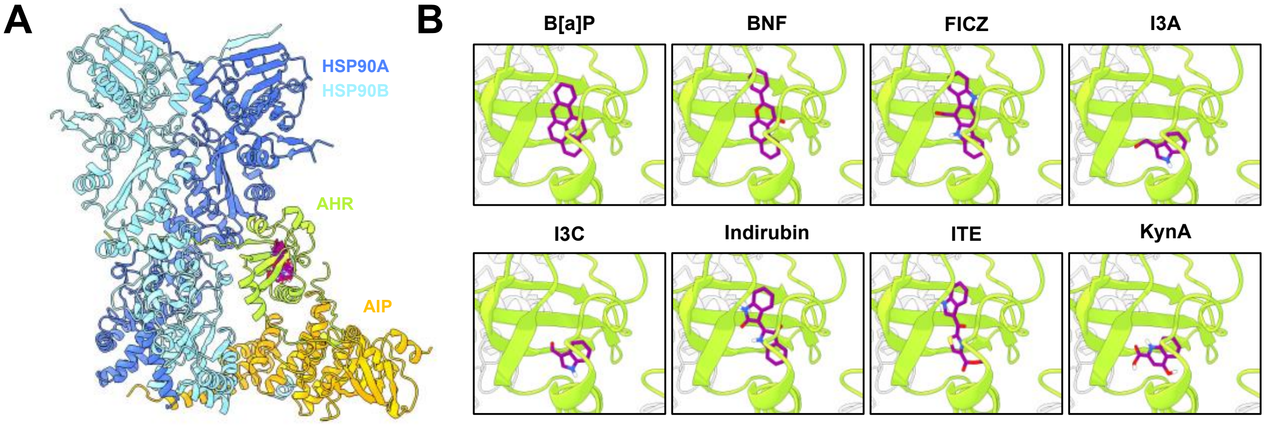

Figure S2. Local docking of AHR agonists within the LBP of AHR using the docking algorithm Vina. (A) Predicted conformations of the AHR agonists (B[a]P, BNF, FICZ, I3A, I3C, Indirubin, ITE, KynA) in the structural model of the indirubin-bound AHR complex. (B) Close-up view of the best poses of AHR agonists in the LBP of the AHR, predicted by Vina.

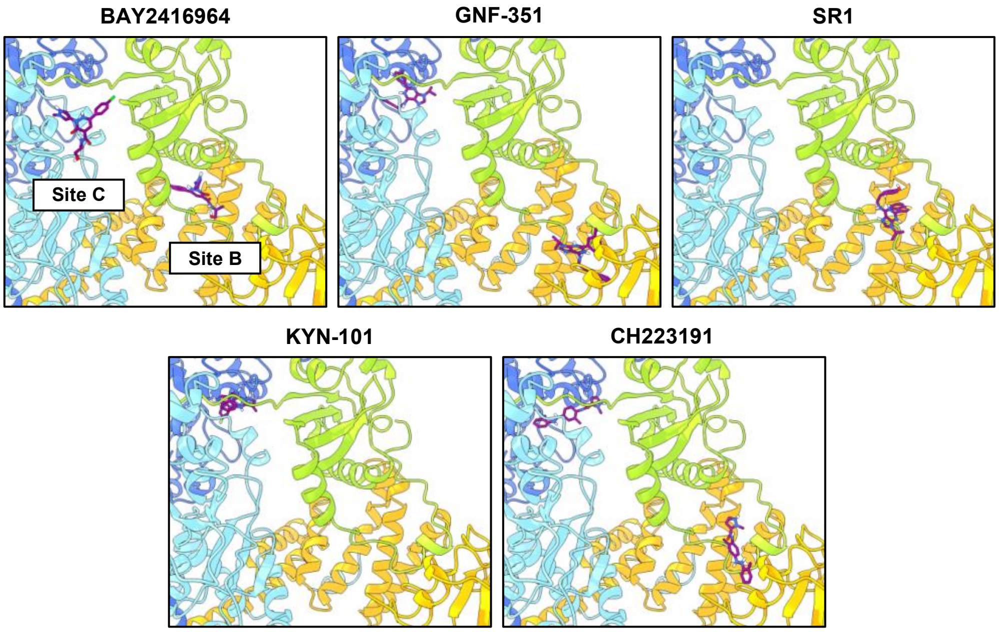

Figure S3. Local docking of AHR antagonists to sites B and C with Vina. The best conformation of each AHR antagonist (BAY2416964, GNF-351, SR-1, KYN-101 and CH223191) in the two sites is shown. The absence of a compound within site B or site C indicates low scores for the predicted poses (score cut-off: 10 poses within 2 kcal/mol of the best-scoring pose).

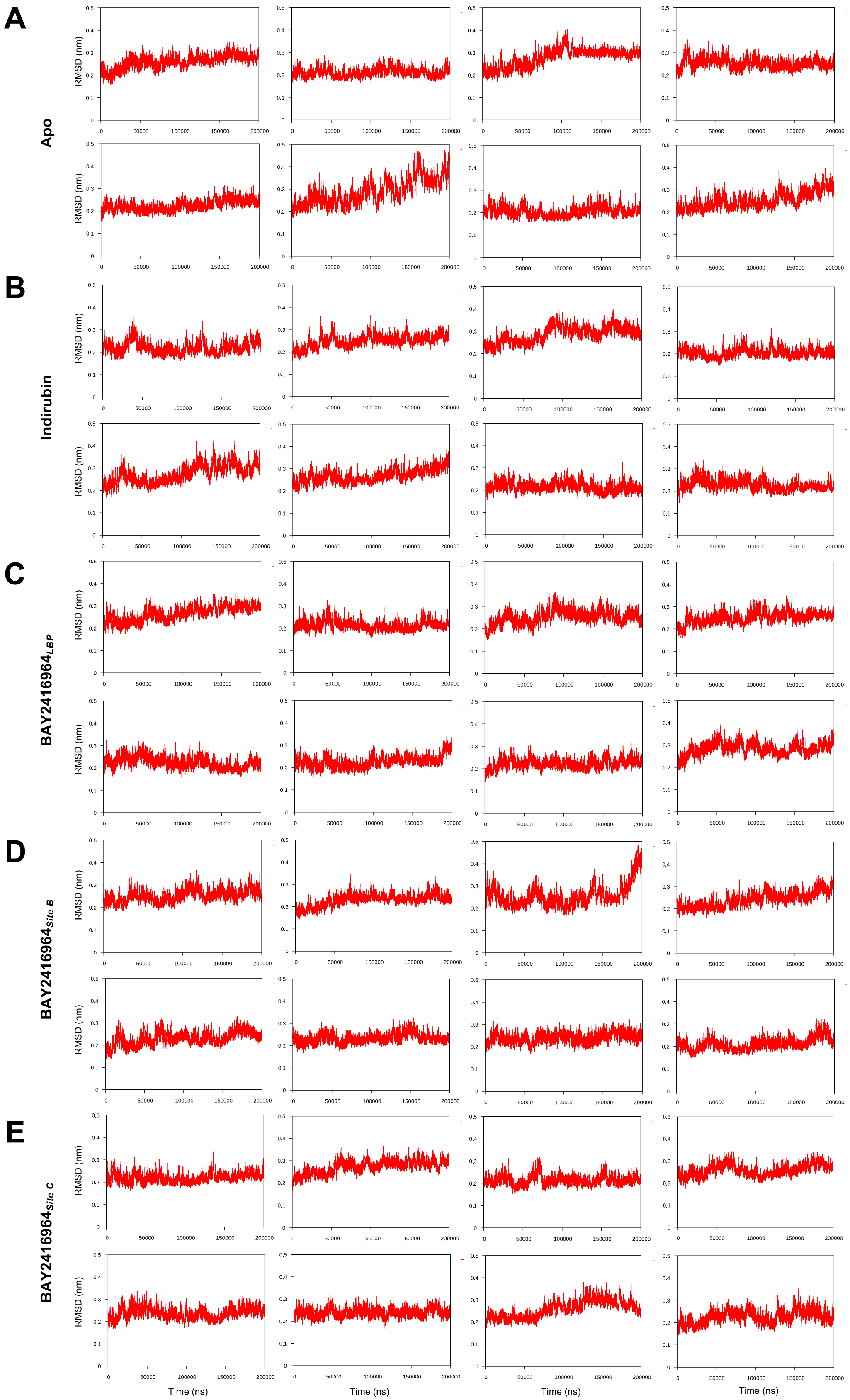

Figure caption on next page

Figure S4. Backbone RMSD of the AHR complex (excluding AIP residues 2-165) across all MD systems and simulations after superimposition to itself. In total, 8 molecular dynamics trajectories of 200 ns each were carried out for each system (Apo (A), Indirubin (B), BAY2416964 in the LBP (C), BAY2416964 in site B (D), BAY2416964 in site C (E)).
